# Accessing Maternal Health Services in Eastern Burma

**DOI:** 10.1371/journal.pmed.0050250

**Published:** 2008-12-23

**Authors:** Macaya Douoguih

## Abstract

Macaya Douoguih discusses a retrospective household survey that characterized the relationships between access to care, health status, and human rights violations in eastern Burma.

Burma is a country that has endured decades of war and civil conflict. The State Peace and Development Council, the current military regime that has governed the country for decades, has had ongoing conflicts with ethnic minority groups. Specific policies of the State Peace and Development Council have resulted in forced labor, forced relocation, torture, killings, and deliberate destruction of food supplies [1–3], largely targeted at ethnic minorities within the country [4]. Over one million Burmese are estimated to have been displaced since 1996 [5], and about half have been unable to resettle or return to their homes. The eastern border of the country bears the largest burden of internally displaced persons in the country [5].

Accurate measures of health are critical to address the high morbidity and mortality in this vulnerable population. However, there is a wide variation in reported estimates of health indicators. For example, the World Health Organization reports a maternal mortality ratio of 360 per 100,000 live births [6]; in 2006 UNICEF reported an under-five mortality rate of 104 per 1,000 live births and an infant mortality rate of 74 per 1,000 live births [7]. In contrast, surveys conducted by the Back Pack Health Worker Team—a community-based organization providing health care to internally displaced persons in active conflict zones (“black zones”)—give very different results. The team's surveys estimate maternal mortality as 1,000–1,200 per 100,000 live births [4] and infant mortality and under-five mortality as 91 per 1,000 live births and 221 per 1,000 live births, respectively [4].

**Linked Research Article**

This Perspective discusses the following new study published in *PLoS Medicine*:

Mullany LC, Lee CI, Yone L, Paw P, Oo EKS, et al. (2008) Access to essential maternal health interventions and human rights violations among vulnerable communities in eastern Burma. PLoS Med 5(12): e242. doi:10.1371/journal.pmed.0050242

Luke Mullany and colleagues examine access to essential maternal health interventions and human rights violations within vulnerable communities in eastern Burma.

## A New Study

Increased adverse health outcomes have been associated with human rights violations in multiple settings [4,8,9]. In eastern Burma, there are limited data on health demographics and patterns of health care delivery. In a new paper published in this issue of *PLoS Medicine*, Luke Mullany and colleagues present results of a retrospective household survey to better characterize the relationships between access to care, health status, and human rights violations in four states in eastern Burma [10]. This study was done in the context of a larger project: The Mobile Obstetric Maternal Health Workers Project, a pilot study to demonstrate whether the introduction of a mobile health system will improve access to maternal health services among the internally displaced in 12 communities in four eastern states [11]. The new *PLoS Medicine* paper reports quantitative baseline data on access to select health services and exposure to human rights violations in project sites [10].

Information was collected on 2,889 women of reproductive age, including demographics, past obstetric history and type of antenatal care received, family planning, and pattern of contraceptive use. Data were also collected on exposure to human rights violations, defined as forced labor, soldier attacks, destruction of food supplies, landmine injuries, and forced displacement [12]. Researchers screened a subset of participants for anemia, malaria parasitemia, and malnutrition. Ninety-seven percent of respondents represented four ethnic groups: Karen, Karenni, Mon, and Shan. Sixty-one percent of women screened were anemic; 19.3% of women had mid-upper-arm circumference measurements below 22.5 cm (a surrogate for moderate malnutrition); and 7.4% were parasitemic (with Plasmodium falciparum). Overall recall rates of receiving tetanus toxoid, iron and folate supplementation, antihelminth medications, and vitamin A were low in all communities.

Most births took place in the home, though the rates of deliveries by skilled attendants were higher in Mon and Shan areas, because women were able to travel to facilities across the border in Thailand. Mon and Shan communities were also more likely to have more than four antenatal care visits, access to postnatal care at a hospital, and access to contraception than women from Karen and Karenni communities.

The prevalence of human rights violations reported varied among the states, as did the types of exposures. There were virtually no reports of human rights violations in the Mon area, but there were high rates of all violations in the Shan area. In Karen communities, who live in non-ceasefire zones, participants who reported forced relocation had nearly three times increased risk of anemia, and those reporting loss of food security had ten times the risk. Respondents who reported forced relocation also were almost six times less likely to receive any antenatal care interventions.

## Strengths and Limitations of the Study

Mullany and colleagues are a strong, ethnically representative, collaborative research team who should be commended for their commitment and capacity to work in a profoundly challenging environment. They conducted a survey in a short timeframe and obtained a very high response rate (99.1%). The inclusion of ceasefire and non-ceasefire regions will yield valuable insight into the influence that different exposure patterns to conflict have on health service delivery and outcomes.

Their study is prone to selection bias because communities participating in the Mobile Obstetric Maternal Health Workers Project were selected based on the stability of the area, proximity to the Thai border, and existing health facilities and programs. The project sites may not be representative of the larger eastern Burmese population, limiting the generalizability of the study results. The correlation between forced relocation and poorer access to health, and between forced relocation and anemia, appears to be strong. However, some potential confounders to consider include concurrent human rights violations, variations in proximity to health services, and other conditions that could contribute to anemia (such as pregnancy, recent obstetric complications, helminth infection, and underlying hemoglobinopathies).

## Implications

The baseline data from this new study provide useful information on access to care and health indicators, which will help to prioritize unmet needs. There is no question that an increase in access to services is desperately needed to improve health in this region. This study lays the foundation for an innovative community-based mobile health system that could greatly enhance the health of communities in eastern Burma. Future considerations should include how to sustain effective health programs, an inherent challenge in eastern Burma and other conflict settings.

## References

[pmed-0050250-b001] Refugees International (2006). Ending the waiting game: Strategies for responding to internally displaced people in Burma. http://www.refugeesinternational.org/sites/default/files/EndingtheWaitingGame.pdf.

[pmed-0050250-b002] Lopes Cardozo B, Talley L, Burton A, Crawford C (2004). Karenni refugees living in Thai-Burmese border camps: Traumatic experiences, mental health outcomes, and social functioning. Soc Sci Med.

[pmed-0050250-b003] Petersen HD, Worm L, Olsen MZ, Ussing B, Hartling OJ (2000). Human rights violations in Burma/Myanmar. A two year follow-up examination. Dan Med Bull.

[pmed-0050250-b004] Back Pack Health Worker Team (2006). Chronic emergency: Health and human rights in eastern Burma. http://burmalibrary.org/docs3/ChronicEmergencyE-ocr.pdf.

[pmed-0050250-b005] Thailand Burma Border Consortium (2006). Internal displacement in Eastern Burma, 2006 survey. http://www.tbbc.org/idps/report-2006-idp-english.pdf.

[pmed-0050250-b006] World Health Organization (2006). World Health Statistics 2006. http://www.who.int/whosis/whostat2006_mortality.pdf.

[pmed-0050250-b007] UNICEF (2006). At a glance: Myanmar. http://www.unicef.org/infobycountry/myanmar_statistics.html.

[pmed-0050250-b008] Lee TJ, Mullany LC, Richards AK, Kuiper HK, Maung C (2006). Mortality rates in conflict zones in Karen, Karenni, and Mon states in eastern Burma. Trop Med Int Health.

[pmed-0050250-b009] Grandesso F, Sanderson F, Kruijt J, Koene T, Brown V (2005). Mortality and malnutrition among populations living in South Darfur, Sudan: Results of 3 surveys, September 2004. JAMA.

[pmed-0050250-b010] Mullany LC, Lee CI, Yone L, Paw P, Oo EKS (2008). Access to essential maternal health interventions and human rights violations among vulnerable communities in eastern Burma. PLoS Med.

[pmed-0050250-b011] Mullany LC, Lee CI, Paw P, Shwe Oo EK, Maung C (2008). The MOM Project: Delivering maternal health services among internally displaced populations in eastern Burma. Reprod Health Matters.

[pmed-0050250-b012] Mullany LC, Richards AK, Lee CI, Suwanvanichkij V, Maung C (2007). Population-based survey methods to quantify associations between human rights violations and health outcomes among internally displaced persons in eastern Burma. J Epidemiol Community Health.

